# Beyond walking: gait context and demands shape arousal and valence evoked by observation in Parkinson’s disease

**DOI:** 10.3389/fneur.2026.1727828

**Published:** 2026-03-23

**Authors:** Martina Putzolu, Elisabetta Sarasso, Elisa Canu, Sara Terranova, Alessandro Botta, Susanna Mezzarobba, Massimo Filippi, Federica Agosta, Laura Avanzino, Elisa Pelosin

**Affiliations:** 1 Department of Neuroscience, Rehabilitation, Ophthalmology, Genetics and Maternal Child Health, University of Genoa, Genoa, Italy; 2 IRCCS Azienda Ospedaliera Metropolitana, Genoa, Italy; 3 Neuroimaging Research Unit, Division of Neuroscience, IRCCS San Raffaele Scientific Institute, Milan, Italy; 4 Neurotech Hub, Vita-Salute San Raffaele University, Milan, Italy; 5 Department of Experimental Medicine, Section of Human Physiology, University of Genoa, Genoa, Italy; 6 Department of Life Sciences, University of Trieste, Trieste, Italy; 7 Neurorehabilitation Unit, IRCCS San Raffaele Scientific Institute, Milan, Italy; 8 Neurophysiology Service, IRCCS San Raffaele Scientific Institute, Milan, Italy

**Keywords:** action observation, emotional processing, environmental context, gait, Parkinson’s disease, task demand

## Abstract

**Introduction:**

Gait disturbance is a hallmark of Parkinson’s disease (PD) that worsens under complex conditions and might change according to environmental context. In this study, we aimed to determine how observing gait demands and context affects arousal and valence in PD.

**Methods:**

Fifty PD participants and 50 healthy subjects (HS) completed a questionnaire evaluating arousal and valence of gait observation. This consisted of 36 videos showing a person walking, which were categorized based on two factors: (i) context: natural or built environment; and (ii) walking demand: Low-Demand (LD, e.g., usual walking along a street); Moderate-Demand (MD, e.g., walking on narrow surfaces); High-Demand (HD, e.g., walking on heightened surfaces).

**Results:**

Arousal scores increased significantly according to environmental context (natural > built environment) and task complexity (HD > MD > LD). Both groups reported a negative valence for demanding conditions, a positive valence for LD in natural environments, and a neutral valence for LD in built environments. When comparing PD and HS, valence attribution was similar, whereas PD participants reported higher arousal than controls.

**Discussion:**

The overall higher arousal levels observed in PD patients compared with HS may reflect a heightened emotional reactivity to walking-related challenges. This study will enhance our understanding of gait-related difficulties in PD.

## 1 Introduction

Gait disturbances are a major disabling sign of Parkinson’s disease (PD) that can manifest as continuous (e.g., reduced step length and gait speed, lower arm oscillation, and increased gait variability) as well as episodic, such as freezing of gait (FOG) (1). Challenging walking conditions worsen gait disorders, raising fall risk (2), and may also influence emotional reactions (3). In turn, emotions might affect gait pattern, with evidence showing that unpleasant emotional states such as sadness, fear, or anger can negatively alter spatiotemporal gait parameters (4–6). For example, early evidence in healthy subjects (HS) indicates that negative emotions may lead to slower gait speed, increased flexion of neck and trunk, and restricted limb movement, whereas positive emotions enhance walking velocity, range of motion, and trunk extension (7, 8).

In PD, negative emotional state, particularly stressful and anxious circumstances, can exacerbate motor impairments, including tremor, and gait disturbances (e.g., FOG) throughout the disease progression (9–12). Conversely, positive emotions may enhance motor performance, for instance increasing walking speed (7). Furthermore, depression and anxiety, which are common in individuals with PD can lead to behavioral changes that further contribute to the complexity of gait disturbances (13).

Actions and behaviors that we perform and observe in everyday life are embedded within contexts that have emotional significance, further shaped by individual past experiences (14). Movements are perceived as part of a broader context that includes other individuals, objects, and their relationship, rather than in isolation. Action understanding depends not only on motor performance but also on the environment in which the action takes place (14–17). Moreover, research shows that walking in nature reduces stress while enhancing positive emotions and attention compared to a built environment (i.e., urban or indoor scenarios) (18).

Arousal and valence are commonly used to investigate emotional responses when observing figures or actions. Specifically, arousal reflects the intensity of an emotion, whereas valence indicates the positive or negative content of an emotion (19). These emotional dimensions may influence task performance (such as action readiness and motor flexibility, reaction times, and motor resonance) and may, in turn, be modulated by motor tasks themselves (14, 19–22). For example, in individuals with PD and FOG, gait initiation can be influenced by the emotional valence of visual stimuli, with a longer reaction time and a shorter step size in response to an unpleasant image (22). Compared to HS, PD patients usually report impaired emotional responses regarding arousal, valence, and emotion recognition (23).

This study aims to determine the effects of gait context and demands on emotional responses in patients with PD compared to age-matched HS. We hypothesized that: (i) the environmental context and demands of gait conditions will influence perceived arousal and valence, with higher arousal and valence in natural environments, higher arousal and more negative valence scores in challenging gait situations, and lower arousal and more positive valence (or higher valence) in low-demand condition of walking; (ii) individuals with PD will differ from HS, exhibiting altered arousal and valence scores.

## 2 Method

### 2.1 Subjects

Fifty individuals with PD and 50 HS participated in this study, approved by the institutional ethical committee of the University of Genova (CERA 2023/02). Written informed consent was obtained from all participants prior to the experimental session, and the study was conducted adhering to the Declaration of Helsinki standards.

Participants with PD were recruited from the outpatient service of the Movement Disorders Clinic at the IRCCS Policlinico San Martino hospital (Genova), while HS were recruited via word of mouth from subjects unrelated to the patient population. Inclusion criteria for PD participants were: (i) diagnosis of idiopathic PD, according to the United Kingdom Parkinson’s Disease Society Brain Bank criteria (24); (ii) Hoehn and Yahr stage I–III (25); (iii) stable antiparkinsonian medication for at least 1 month prior to the study. All participants were required to meet the inclusion criteria of being aged less than 85 years. Common exclusion criteria were: (i) presence of cognitive impairments [Montreal Cognitive Assessment score *≤*17 (26)]; and/or other health conditions that might interfere with participation (e.g., severe visual or upper limb deficits).

### 2.2 Experimental design and procedure

Clinical data, including disease duration (i.e., years since PD diagnosis) and motor severity (assessed using part III of the Movement Disorders Society—Unified Parkinson’s Disease Rating Scale, MDS-UPDRS III) (27), and the presence and severity of depression and anxiety [measured using the Beck Depression Inventory-II (28) and the Hamilton Anxiety Rating Scale (29), respectively] were collected during routine clinical visits. Subsequently, all participants received a link to complete a custom-designed online questionnaire. The questionnaire first collected demographic information (age, gender, and years of education), and then assessed emotional responses, namely the arousal and valence, evoked by observing gait performed in various contexts and under different task demands.

Specifically, a total of 36 publicly available videos were selected from the YouTube platform, edited to align with the aim of this study, and categorized based on two factors: (i) Context: natural (NAT) or built (BUILT) environment; and (ii) Walking demand: Low-Demand (LD, e.g., usual walking along a street); Moderate-Demand (MD, e.g., walking on narrow surfaces); and High-Demand (HD, e.g., walking on heightened surfaces).

Each video included an individual walking (with the face not visible) shown from various perspectives (third- or first-person; full description in Supplementary Table 1s). Videos lasted approximately 5 s each and were presented once, in a pseudo-randomized order across environmental context and task demands, where stimuli expected to elicit high arousal were separated by at least one lower-arousal stimulus. Importantly, the pseudo-random sequence was fixed and identical for all participants, with no individual randomization of presentation order.

**Figure 1:**
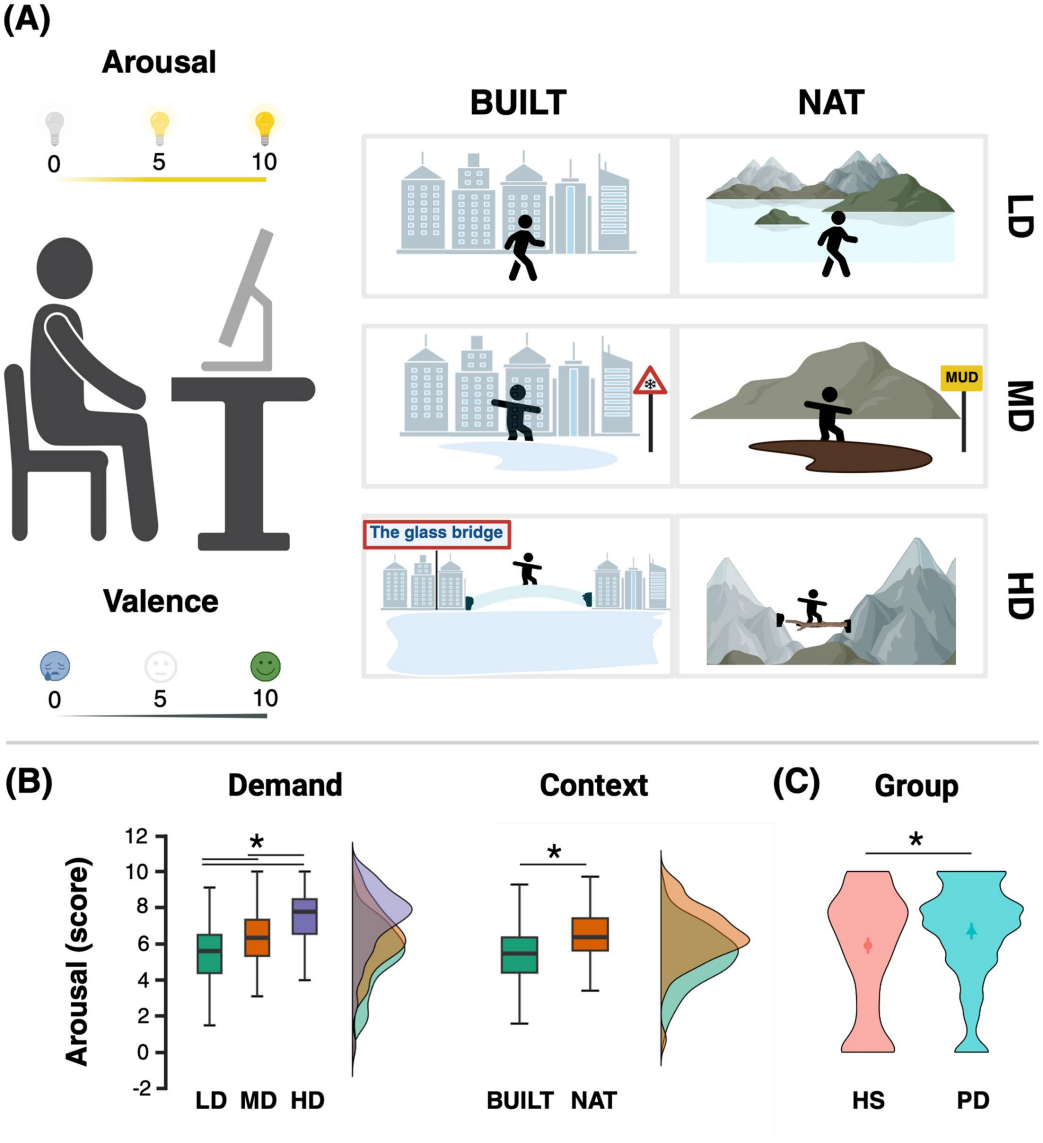
**(A)** Setup of the questionnaire addressing arousal and valence evoked by observing gait performed in various contexts and with different task demands; **(B)** arousal scores comparison in LD, MD, and HD conditions (gait demand) and in built and nature conditions (gait context), separately; **(C)** between-group arousal scores comparison (HS vs. PD). BUILT, walking in a built environment; NAT, walking in natural scenarios; LD, walking with a low demand; MD, walking with moderate demand; HD, walking with high demand; HS, healthy subjects; PD, Parkinson’s disease patients. Created in BioRender (Pelosin, 2026) https://BioRender.com/2789r6k and JASP.

After viewing each video, participants rated their perceived arousal and the valence using a Numeric Rating Scale. Arousal, defined as “the physical or mental activation” resulting from observing the presented gait (considering context), was rated on a scale from 0 (“no activation”) to 10 (“maximum activation”). Valence, defined as the pleasantness or unpleasantness of the observed gait (including its context), was scored from 0 (“totally unpleasant”) to 10 (“totally pleasant”), with a midpoint score of 5 indicating “neither pleasant nor unpleasant.” The experimental session about the evaluation of arousal and valence of gait observation is depicted in Figure 1A.

For PD participants, the entire protocol was conducted during the “ON” phase of their medication cycle.

Since the High-Demand condition included walking in height-related contexts, we administered the Visual Height Intolerance Severity Scale (VHISS) (30) to evaluate the presence of VHI as a potential influencing factor.

### 2.3 Statistical analysis

Arousal and valence ratings were analyzed using a Linear Mixed-effects Model (LMM). Separate models were fitted for arousal and valence scores. Valence scores were centered on the neutral midpoint (i.e., neutral score = 0) prior to the execution of the LMM, with negative values indicating unpleasant ratings and positive values indicating pleasant ratings. In each model, Group (PD, HS), Context (natural, built), and Demand (LD, MD, HD), demographic (Age, Sex), and presence of visual height intolerance were included as fixed effects. Random intercepts were included for participants and video stimuli. Effect sizes were quantified using semi-partial R^2^ values derived from the LMMs. Where appropriate, *post hoc* comparisons were conducted using Bonferroni-adjusted *p*-values.

For the PD group, correlation analyses were conducted to explore possible associations of each emotional measure (arousal and valence), separately, with clinical (i.e., MDS-UPDRS sub-items related to emotion functioning—items 3.2 and 3.13) and neuropsychological measures. For this purpose, mean arousal and valence scores were computed for each participant and condition. Given the different measurement properties of arousal (unipolar) and valence (bipolar with a neutral midpoint), scores were standardized (*z*-scores) before correlation analyses to improve comparability across scales.

Statistical analyses were performed using JASP (Version 0.19.1) and BlueSky Statistics (Version 10.3.4). The significance level was set at *p* < 0.05.

## 3 Results

Two PD patients were excluded due to incomplete data. Using a matching procedure, forty-eight HS were matched with 48 PD participants based on age. Demographics of both groups and the clinical characteristics of PD participants are provided in Table 1. Regarding visual height intolerance, twenty-two (46%) HS and 20 (42%) PD participants reported having experienced VHI. Detailed statistical results of the LLMs are reported in Supplementary File 2s.

**Table 1:** Demographics of both groups and clinical characteristics of PD participants.

Variable	PD (*n* = 48)	HS (*n* = 48)
Age (years)	67 [60–71]	72 [63–74]
Gender (F)	14 (29%)	29 (60%)
Education (years)	13 [13–17.25]	11 [7.25–14.75]
Disease duration (years)	4 [1–8]	–
MDS-UPDRS-part III (score)	20 [10–28]	–
MDS-UPDRS-item 3.2	1 [1–2]	–
MDS-UPDRS-item 3.13	1 [0–1]	–
BDI-II (score)	8 [5–12]	–
HAM-A (score)	6.5 [2.75–9.25]	–

Values are presented as median [interquartile range], and as percentage. PD, Parkinson disease; HS, healthy subjects; F, female; MDS-UPDRS, Movement Disorder Society-Unified Parkinson’s Disease Rating Scale; BDI-II, Beck Depression Inventory II; HAM-A, Hamilton Anxiety Rating Scale.

### 3.1 Arousal

The linear mixed-effects model revealed significant main effects of Demand [*F*(2, 30.15) = 30.22, *p* < 0.0001, semi-partial *R*^2^ = 0.667], Context [*F*(1, 30.01) = 7.47, *p* = 0.0104, semi-partial *R*^2^ = 0.199], Group [*F*(1, 93.50) = 7.14, *p* = 0.0089, semi-partial *R*^2^ = 0.071], and VHI [*F*(1, 92.73) = 4.70, *p* = 0.0327, semi-partial *R*^2^ = 0.048]. In addition, significant interactions emerged for Demand × Context [*F*(2, 30.01) = 14.58, *p* < 0.0001, semi-partial *R*^2^ = 0.493], and Demand × VHI [*F*(2, 3309.16) = 7.40, *p* = 0.0006, semi-partial *R*^2^ = 0.005]. Age and Sex did not show significant main effects, and no additional interaction effects were observed (all *p* > 0.05).

Bonferroni-adjusted pairwise comparisons showed that arousal scores significantly increased with task Demand (Figure 1B), with HD conditions eliciting higher arousal than both MD and LD conditions (HD vs. LD, *p* < 0.0001; HD vs. MD, *p* = 0.0020), and MD conditions eliciting higher arousal than LD (*p* = 0.0013).

Arousal was also significantly higher during observation of gait in NAT compared with BUILT environments (*p* = 0.0066; Figure 1B), higher in the PD group compared with HS (*p* = 0.0075; Figure 1C), and higher in participants reporting VHI compared with those without VHI (*p* = 0.0302).

*Post hoc* analyses of the Demand × Context interaction indicated that, in BUILT environments, both HD and MD conditions elicited higher arousal than LD (*p* < 0.0001), whereas in NAT environments arousal was significantly higher in HD compared with MD (*p* = 0.0072). In addition, observation of gait in NAT environments elicited higher arousal than BUILT environments in the LD condition (*p* < 0.0001).

Regarding the Demand × VHI interaction, participants reporting VHI showed significantly higher arousal than those without VHI, specifically during HD conditions (*p* = 0.0025), whereas no significant differences were observed in LD or MD conditions.

### 3.2 Valence

In terms of valence, both groups reported unpleasant ratings for demanding conditions regardless of context, pleasant ratings for the LD condition in natural environments, and neutral scores for the LD condition in built environments.

The linear mixed-effects model for valence ratings revealed significant main effects of Demand [*F*(2, 30.08) = 42.64, *p* < 0.0001, semi-partial *R*^2^ = 0.739] and Context [*F*(1, 30.00) = 16.65, *p* = 0.0003, semi-partial *R*^2^ = 0.357]. A significant Demand × VHI interaction was also observed [*F*(2, 3310.14) = 14.45, *p* < 0.0001, semi-partial *R*^2^ = 0.009]. No significant main effects or interactions involving Group, Age, and Sex were found (all *p* > 0.05).

Bonferroni-adjusted *post hoc* comparisons showed that valence scores were significantly higher in LD conditions compared with both MD and HD conditions (*p* < 0.0001), indicating a progressive reduction in positive affect with increasing task demand. Valence ratings were also significantly higher in NAT compared with BUILT environments (*p* < 0.0001).

*Post hoc* analyses of the Demand × VHI interaction revealed that, under HD conditions, participants without VHI reported significantly higher valence scores compared with those experiencing VHI (*p* = 0.0006), whereas no significant differences emerged in LD or MD conditions.

### 3.3 Correlations

Within the PD group, correlational analyses were conducted between standardized (z-score) condition-averaged arousal and valence scores and neuropsychological or clinical measures related to emotional functioning (MDS-UPDRS items 3.2 and 3.13). No significant correlations were observed for either arousal or valence scores with these clinical or neuropsychological measures (all *p* > 0.05).

## 4 Discussion

In this study, we examined how observing gait in various environmental contexts and under different task demands influences arousal and valence perception in individuals with PD compared to HS. Specifically, we compared responses to videos depicting walking in either natural or built environments, under low (e.g., usual walking), moderate (e.g., requiring balance), and high (e.g., heightened settings) task demand conditions.

As hypothesized, arousal increased in response to both the natural context and the higher task demand, suggesting that these factors enhance emotional activation. Although natural environments are often associated with stress reduction and positive affect (31), the higher arousal observed during gait observation in natural contexts could be attributed to specific characteristics of the observed gait scenario. In particular, natural walking environments included more variable and less predictable gait-related conditions than built walking environments, including uneven terrain or narrow paths or potentially demanding elements, which could have increased attentional and emotional engagement when observing gait (32–34). Accordingly, the higher arousal observed in the natural compared to built environments should not be interpreted as in contrast to the positive effects of nature reported previously (31), but rather as reflecting the emotional demands associated with observing gait in more complex and less predictable situations. Importantly, the observed arousal effects reflect emotional responses specifically related to evaluating gait under varying task demands, rather than general emotional states associated with passive viewing of natural environments.

In line with our hypothesis, walking in built environments was generally rated as neutral, walking in natural environments as pleasant, and walking under high-demand conditions as unpleasant.

Between-group comparisons revealed a similar ability to evaluate valence, although individuals with PD reported higher arousal levels compared to HS. Importantly, this group difference in arousal was not explained by demographic factors, as neither age nor sex showed a significant effect in the LMM, and no significant associations emerged between arousal ratings and clinical or neuropsychological tests within the PD group. However, although PD participants showed higher overall arousal levels, the effects of gait context and task demand on arousal did not significantly differ between groups. These results suggest that the contextual and task-related modulation of emotional responses during gait observation may rely on general motor resonance and action observation mechanisms, which appear to remain relatively preserved in our PD sample (35–38).

Findings regarding valence contrast with previous research suggesting impaired emotion recognition in PD, particularly in individuals with cognitive impairment (23, 39). One possible explanation is that our PD sample excluded participants with mild cognitive impairment, who are more likely to show emotional processing deficits. Thus, the preserved valence recognition observed in our study may reflect the relatively intact cognitive and emotional functioning of our sample. Conversely, the overall higher arousal observed in PD participants may indicate increased awareness of motor deficits. This finding is in line with recent neuroimaging studies showing increased activity in cognitive and associative networks in PD during a simple motor task used as a proxy of walking in an MRI scan (37).

Finally, visual height intolerance further modulated emotional responses to gait observation. Indeed, VHI was associated with higher arousal and lower valence during high-demand gait observation, highlighting the role of individual factors in shaping emotional reactions to challenging situations.

Overall, our results align with the inverted-U hypothesis (40), which posits that performance enhances with increasing arousal levels, up to an optimal threshold, after which further arousal leads to decreased performance. A recent work (10) suggests that the higher arousal observed in PD might sometimes be beneficial, for example in life-threatening circumstances (41, 42), in social contexts due to increased motivation (43), or when individuals are aware of being observed (i.e., the “Hawthorne effect”) (44). However, when arousal exceeds the optimal threshold, it may disrupt gait performance, thus requiring strategies to reduce demands through compensatory mechanisms (e.g., facilitating relaxation or decreasing social pressure and negative feelings).

Several limitations should be considered when interpreting the findings of the present study. First, the sample size was relatively modest, and neuropsychological data were not collected for HS, limiting between-group comparisons. Second, information regarding participants’ previous experiences in similar real-world gait situations (e.g., falls or near-falls in natural or complex environments) was not collected. Such experiences may modulate threat perception and, consequently, influence emotional responses during gait observation. Third, variability in video content (e.g., “actors,” scenarios, and perspectives) and presentation may have introduced uncontrolled factors affecting arousal and valence ratings. Specifically, the inter-stimulus interval was self-paced (no fixed ISI), which may have allowed residual emotional responses to carry over between trials. Furthermore, the use of the same predetermined pseudo-random sequence for all participants, rather than individualized randomization, may have introduced systematic order effects. Nevertheless, the use of videos offers high ecological validity by reflecting the variability of real-life gait scenarios. However, this approach offers less experimental control than standardized stimuli (e.g., vignettes).

Finally, the lack of online physiological measurements (e.g., galvanic skin response) represents a limitation that should be addressed in future studies.

In conclusion, this study provides novel insights into how contextual and task-related factors influence the emotional perception of gait in PD. Our findings suggest that arousal and valence ratings during gait observation are sensitive to environmental cues and task complexity, and that PD patients may exhibit overall supraoptimal arousal. Future studies should include larger cohorts and motor assessment to better determine whether the heightened arousal response in individuals with PD is functionally beneficial or detrimental.

Identifying individual arousal ranges that optimize gait performance, as well as thresholds beyond which excessive arousal becomes detrimental, could inform personalized “altering the mental state” strategies (10). Such insights may contribute to interventions aimed at enhancing walking confidence and reducing fear in PD, thereby supporting targeted gait rehabilitation approaches.

## Data Availability

The datasets presented in this study can be found in online repositories. The names of the repository/repositories and accession number(s) can be found at: Open Science Framework repository, https://osf.io/qcj23/?view_only=89963499d2f64b44b6905a6f4dfd8175.
