# Supplementary material for: Beyond walking: gait context and demands shape arousal and valence evoked by observation in Parkinson’s disease
Source: Front Neurol. 2026 Mar 23;17:1727828. doi: 10.3389/fneur.2026.1727828 (PMC13051502; doi:10.3389/fneur.2026.1727828)
Supplement: Supplementary file 1 [file Supplementary_file_1.pdf]

Table 1s. Description of the 36 videos included in the questionnaire.

ACTION	DEMAND	CONTEXT	ACTOR SEX	VIEW	PERSPECTIVE
Walking in a hall	LD	BUILT	M	POST	3°
Walking in a room with neutral background	LD	BUILT	F	LAT	3°
Walking in a pedestrian road	LD	BUILT	M	POST	3°
Walking down the sidewalk	LD	BUILT	F	ANT	3°
Walking down the sidewalk with trekking clothes	LD	BUILT	M	LAT	3°
Walking down a corridor	LD	BUILT	F	POST	3°
Walking down a crowded sidewalk	LD	BUILT	M	POST	3°
Crossing the zebra crossing	LD	BUILT	M	POST	3°
Walking in a city park	LD	BUILT	M	LAT	3°
Walking in the woods	LD	NATURE	M	POST	3°
Walking in the mountains	LD	NATURE	M	POST	3°
Walking in the woods	LD	NATURE	M	HIGH	3°
Walking in the countryside	LD	NATURE	M	LAT	3°
Walking on a boardwalk	LD	NATURE	F	POST	3°
Walking in the mountains	LD	NATURE	M	POST	3°
Walking in the snow with snowshoes	LD	NATURE	UN	FEET	1°
Walking in the snow with trekking boot	LD	NATURE	UN	FEET	1°
Walking in the countryside	LD	NATURE	F	POST	3°
Walking on ice	MD	NATURE	UN	FEET	1°
Walking barefoot in the mud	MD	NATURE	UN	FEET	1°
Walking with difficulty in a swamp	MD	NATURE	M	POST	3°
Walking on coal	MD	BUILT	M	ANT	3°
Crossing the street among scooters	MD	BUILT	M	POST	3°
An elderly woman crossing the street in traffic	MD	BUILT	F	HIGH	3°
Walking on rocks barefoot	MD	NATURE	M	POST	3°
Walking in the desert uphill	MD	NATURE	UN	FEET	1°
Tandem walking	MD	NATURE	UN	FEET	1°
Walking on a glass bridge	HD	BUILT	UN	FEET	1°
Walking on Tibetan bridge	HD	NATURE	F	POST	3°
Walking on a suspended wooden log	HD	NATURE	M	LAT	3°
Walking on a glass bridge with socs	HD	BUILT	UN	FEET	1°
Walking on Tibetan bridge in the wind	HD	NATURE	M	LAT	3°
Walking on Tibetan bridge	HD	NATURE	F	ANT	3°
Walking on a skyscraper	HD	BUILT	F	FEET	1°
Sideways walking on a skyscraper	HD	BUILT	F	FEET	1°
Walking on Tibetan bridge	HD	NATURE	UN	FEET	3°

LD, Low-Demand; MD, Moderate-Demand; HD, High-Demand; M, male; F, female; UN, undefined; POST, posterior view - the person walks away from the camera; ANT, anterior view - the person walks toward the camera; LAT, lateral view - the person is seen walking from the side; HIGH, the person walking is showed from above; FEET, the view focuses exclusively on the feet during walking; 1°, first-person perspective; 3°, third-person perspective

Supplementary File 2s

Results: arousal

Linear Mixed Models

ANOVA Summary

Effect	df	F	p
Age	1, 91.01	1.1293	.2907
Sex	1, 91.01	0.6426	.4248
Group	1, 93.50	7.1430	.0089
Demand	2, 30.15	30.2224	<.0001
Context	1, 30.01	7.4702	.0104
VHI	1, 92.73	4.6999	.0327
Group ∗ Demand	2, 3309.19	1.3201	.2672
Group ∗ Context	1, 3309.08	0.3562	.5506
Demand ∗ Context	2, 30.01	14.5820	<.0001
Demand ∗ VHI	2, 3309.16	7.3997	.0006
Group ∗ Demand ∗ Context	2, 3309.08	0.6645	.5146

Note. Model terms tested with Satterthwaite testMethod.
Note. The following variables are used as random effects grouping factors: 'ID', 'video_id'. *Note*. Type III Sum of Squares

Model summary

Fit statistics

Deviance (REML)	log Lik.	df	AIC	BIC
16269.2711	−8134.6355	20	16309.2711	16432.1763

Note. The model was fitted using restricted maximum likelihood. Please note that models with different fixed effects cannot be compared when REML is used. To use ML, switch 'Test method' to 'Likelihood ratio tests'.

Sample sizes

	Levels of RE grouping factors	
Observations	ID	video_id
3447	96	36

Fixed Effects Estimates

Term	Estimate	SE	df	t	p
Intercept	7.5886	1.2196	92.4028	6.2223	<.0001
Age	−0.0192	0.0180	91.0091	−1.0627	.2907
Sex (1)	−0.1178	0.1469	91.0062	−0.8016	.4248
Group (1)	−0.3998	0.1496	93.4974	−2.6726	.0089
Demand (1)	1.0219	0.1687	30.1582	6.0585	<.0001
Demand (2)	−0.9877	0.1440	30.1489	−6.8587	<.0001
Context (1)	−0.3139	0.1148	30.0089	−2.7332	.0104
VHI (1)	0.3144	0.1450	92.7263	2.1679	.0327
Group (1) ∗ Demand (1)	0.0549	0.0662	3309.2703	0.8300	.4066
Group (1) ∗ Demand (2)	−0.0909	0.0564	3309.0859	−1.6108	.1073
Group (1) ∗ Context (1)	0.0269	0.0450	3309.0828	0.5969	.5506
Demand (1) ∗ Context (1)	0.2744	0.1685	30.0207	1.6286	.1138
Demand (2) ∗ Context (1)	−0.7732	0.1438	30.0106	−5.3751	<.0001
Demand (1) ∗ VHI (1)	0.2259	0.0656	3309.2214	3.4430	.0006
Demand (2) ∗ VHI (1)	−0.1691	0.0559	3309.0851	−3.0228	.0025
Group (1) ∗ Demand (1) ∗ Context (1)	−0.0353	0.0661	3309.0900	−0.5339	.5934
Group (1) ∗ Demand (2) ∗ Context (1)	−0.0411	0.0564	3309.0891	−0.7292	.4659

Note. The intercept corresponds to the (unweighted) grand mean; for each factor with k levels, k - 1 parameters are estimated with sum contrast coding. Consequently, the estimates cannot be directly mapped to factor levels. Use estimated marginal means for obtaining estimates for each factor level/design cell or their differences.

Linear Mixed Models_Demand

ANOVA Summary

Effect	df	F	p
Age	1, 91.01	1.1293	.2907
Sex	1, 91.01	0.6426	.4248
Group	1, 93.50	7.1430	.0089
Demand	2, 30.15	30.2224	<.0001
Context	1, 30.01	7.4702	.0104
VHI	1, 92.73	4.6999	.0327
Group ∗ Demand	2, 3309.19	1.3201	.2672
Group ∗ Context	1, 3309.08	0.3562	.5506
Demand ∗ Context	2, 30.01	14.5820	<.0001
Demand ∗ VHI	2, 3309.16	7.3997	.0006
Group ∗ Demand ∗ Context	2, 3309.08	0.6645	.5146

Note. Model terms tested with Satterthwaite testMethod.

Note. The following variables are used as random effects grouping factors: 'ID', 'video_id'. *Note*. Type III Sum of Squares

Estimated Marginal Means

				95% CI	
Row	Demand	Estimate	SE	Lower	Upper
1	HD	7.3218	0.2521	6.8277	7.8159
2	LD	5.3122	0.2011	4.9181	5.7063
3	MD	6.2657	0.2619	5.7524	6.7791

Note. Results are averaged over the levels of: Sex, Group, Context, VHI.

contrasts

	Estimate	SE	df	95% CI Lower	95% CI Upper	z	p†
HD vs LD	2.0096	0.2612	∞	1.4976	2.5216	7.6924	<.0001
LD vs MD	-0.9535	0.2707	∞	-1.4841	-0.4230	-3.5224	.0013
HD vs MD	1.0561	0.3105	∞	0.4475	1.6647	3.4011	.0020

† P-values are adjusted using Bonferroni adjustment.

Linear Mixed Models_Context

ANOVA Summary

Effect	df	F	p
Age	1, 91.01	1.1290	.2908
Sex	1, 91.49	0.6335	.4281
Group	1, 93.53	7.1677	.0088
Demand	2, 30.18	29.6033	<.0001
Context	1, 30.03	7.3855	.0108
VHI	1, 92.72	4.6967	.0328
Group ∗ Demand	2, 2189.65	1.2052	.2998
Group ∗ Context	1, 2171.39	0.3727	.5416
Demand ∗ Context	2, 30.03	14.4694	<.0001
Demand ∗ VHI	2, 3281.52	7.3813	.0006
Group ∗ Demand ∗ Context	2, 2172.18	0.6607	.5166

Note. Model terms tested with Satterthwaite testMethod.
Note. The following variables are used as random effects grouping factors: 'ID', 'video_id'. *Note*. Type III Sum of Squares

Estimated Marginal Means

				95% CI	
Row	Context	Estimate	SE	Lower	Upper
1	BUILT	5.9861	0.2217	5.5516	6.4206
2	NAT	6.6090	0.1986	6.2198	6.9982

Note. Results are averaged over the levels of: Sex, Group, Demand, VHI.

contrasts

				95% CI			
	Estimate	SE	df	Lower	Upper	z	p†
BUILT vs NAT	−0.6229	0.2292	∞	−1.0722	−0.1737	−2.7176	.0066

† P-values are adjusted using Bonferroni adjustment.

Linear Mixed Models_Group

ANOVA Summary

Effect	df	F	p
Age	1, 91.01	1.1293	.2907
Sex	1, 91.01	0.6426	.4248
Group	1, 93.50	7.1430	.0089
Demand	2, 30.15	30.2224	<.0001
Context	1, 30.01	7.4702	.0104
VHI	1, 92.73	4.6999	.0327
Group * Demand	2, 3309.19	1.3201	.2672
Group * Context	1, 3309.08	0.3562	.5506
Demand * Context	2, 30.01	14.5820	<.0001
Demand * VHI	2, 3309.16	7.3997	.0006
Group * Demand * Context	2, 3309.08	0.6645	.5146

Note. Model terms tested with Satterthwaite testMethod.
Note. The following variables are used as random effects grouping factors: 'ID', 'video_id'. *Note*. Type III Sum of Squares

Estimated Marginal Means

				95% CI	
Row	Group	Estimate	SE	Lower	Upper
1	HS	5.9001	0.2271	5.4550	6.3452
2	PD	6.6997	0.2353	6.2386	7.1609

Note. Results are averaged over the levels of: Sex, Demand, Context, VHI.

contrasts

				95% CI			
	Estimate	SE	df	Lower	Upper	z	p†
HS vs PD	−0.7996	0.2992	∞	−1.3860	−0.2132	−2.6726	.0075

† P-values are adjusted using Bonferroni adjustment.

Linear Mixed Models_VHI

ANOVA Summary

Effect	df	F	p
Age	1, 91.01	1.1293	.2907
Sex	1, 91.01	0.6426	.4248
Group	1, 93.50	7.1430	.0089
Demand	2, 30.15	30.2224	< .0001
Context	1, 30.01	7.4702	.0104
VHI	1, 92.73	4.6999	.0327
Group ∗ Demand	2, 3309.19	1.3201	.2672
Group ∗ Context	1, 3309.08	0.3562	.5506
Demand ∗ Context	2, 30.01	14.5820	< .0001
Demand ∗ VHI	2, 3309.16	7.3997	.0006
Group ∗ Demand ∗ Context	2, 3309.08	0.6645	.5146

Note. Model terms tested with Satterthwaite testMethod.
Note. The following variables are used as random effects grouping factors: 'ID', 'video_id'. *Note*. Type III Sum of Squares

Estimated Marginal Means

				95% CI	
Row	VHI	Estimate	SE	Lower	Upper
1	YES	6.6143	0.2394	6.1452	7.0835
2	NO	5.9855	0.2167	5.5608	6.4102

Note. Results are averaged over the levels of: Sex, Group, Demand, Context.

contrasts

				95% CI			
	Estimate	SE	df	Lower	Upper	z	p†
YES vs NO	0.6288	0.2901	∞	0.0603	1.1973	2.1679	.0302

† P-values are adjusted using Bonferroni adjustment.

Linear Mixed Models_Demand*Context

ANOVA Summary

Effect	df	F	p
Age	1, 91.01	1.1293	.2907
Sex	1, 91.01	0.6426	.4248
Group	1, 93.50	7.1430	.0089
Demand	2, 30.15	30.2224	<.0001
Context	1, 30.01	7.4702	.0104
VHI	1, 92.73	4.6999	.0327
Group ∗ Demand	2, 3309.19	1.3201	.2672
Group ∗ Context	1, 3309.08	0.3562	.5506
Demand ∗ Context	2, 30.01	14.5820	<.0001
Demand ∗ VHI	2, 3309.16	7.3997	.0006
Group ∗ Demand ∗ Context	2, 3309.08	0.6645	.5146

Note. Model terms tested with Satterthwaite testMethod.
Note. The following variables are used as random effects grouping factors: 'ID', 'video_id'. *Note*. Type III Sum of Squares

Estimated Marginal Means

Row	Demand	Context	Estimate	SE	95% CI Lower	95% CI Upper
1	HD	BUILT	7.2823	0.3454	6.6054	7.9592
2	LD	BUILT	4.2251	0.2509	3.7335	4.7168
3	MD	BUILT	6.4506	0.3911	5.6841	7.2172
4	HD	NAT	7.3613	0.3147	6.7446	7.9780
5	LD	NAT	6.3993	0.2509	5.9076	6.8910
6	MD	NAT	6.0808	0.2923	5.5079	6.6538

Note. Results are averaged over the levels of: Sex, Group, VHI.

contrasts

	Estimate	SE	df	95% CI Lower	95% CI Upper	z	p†
BUILT: HD vs LD	3.0572	0.3827	∞	2.3071	3.8073	7.9881	<.0001
BUILT: HD vs MD	0.8317	0.4863	∞	−0.1215	1.7848	1.7102	.6979
BUILT: LD vs MD	−2.2255	0.4244	∞	−3.0574	−1.3936	−5.2435	<.0001
NAT: HD vs LD	0.9620	0.3553	∞	0.2658	1.6583	2.7080	.0541
NAT: HD vs MD	1.2805	0.3857	∞	0.5246	2.0364	3.3202	.0072
NAT: LD vs MD	0.3184	0.3357	∞	−0.3394	0.9763	0.9487	1.0000
HD: BUILT vs NAT	-0.0790	0.4270	∞	-0.9159	0.7579	-0.1850	1.0000
MD: BUILT vs NAT	0.3698	0.4499	∞	−0.5121	1.2517	0.8219	1.0000
LD: BUILT vs NAT	−2.1741	0.3000	∞	−2.7622	−1.5861	−7.2463	<.0001

† P-values are adjusted using Bonferroni adjustment.

Linear Mixed Models_Demand*VHI

ANOVA Summary

Effect	df	F	p
Age	1, 91.01	1.1293	.2907
Sex	1, 91.01	0.6426	.4248
Group	1, 93.50	7.1430	.0089
Demand	2, 30.15	30.2224	<.0001
Context	1, 30.01	7.4702	.0104
VHI	1, 92.73	4.6999	.0327
Group ∗ Demand	2, 3309.19	1.3201	.2672
Group ∗ Context	1, 3309.08	0.3562	.5506
Demand ∗ Context	2, 30.01	14.5820	<.0001
Demand ∗ VHI	2, 3309.16	7.3997	.0006
Group ∗ Demand ∗ Context	2, 3309.08	0.6645	.5146

Note. Model terms tested with Satterthwaite testMethod.
Note. The following variables are used as random effects grouping factors: 'ID', 'video_id'. *Note*. Type III Sum of Squares

Estimated Marginal Means

Row	Demand	VHI	Estimate	SE	95% CI Lower	95% CI Upper
1	HD	YES	7.8621	0.3100	7.2546	8.4697
2	LD	YES	5.4575	0.2619	4.9442	5.9709
3	MD	YES	6.5233	0.3180	5.9000	7.1466
4	HD	NO	6.7815	0.2886	6.2158	7.3472
5	LD	NO	5.1669	0.2397	4.6970	5.6367
6	MD	NO	6.0082	0.2971	5.4259	6.5904

Note. Results are averaged over the levels of: Sex, Group, Context.

contrasts

	Estimate	SE	df	95% CI Lower	95% CI Upper	z	p†
HD: YES vs NO	1.0806	0.3233	∞	0.4470	1.7142	3.3427	.0025
LD: YES vs NO	0.2907	0.3007	∞	−0.2986	0.8799	0.9668	1.0000
MD: YES vs NO	0.5151	0.3231	∞	−0.1182	1.1485	1.5942	.3327

† P-values are adjusted using Bonferroni adjustment.

Results: valence

Linear Mixed Models

ANOVA Summary

Effect	df	F	p
Age	1, 91.00	0.0704	.7914
Sex	1, 91.00	2.7090	.1032
Group	1, 93.95	0.2845	.5950
Demand	2, 30.08	42.6359	< .0001
Context	1, 30.00	16.6459	.0003
VHI	1, 93.03	3.1560	.0789
Group ∗ Demand	2, 3310.17	2.7556	.0637
Group ∗ Context	1, 3310.05	0.5215	.4702
Demand ∗ Context	2, 30.00	1.8906	.1685
Demand ∗ VHI	2, 3310.14	14.4523	< .0001
Group ∗ Demand ∗ Context	2, 3310.05	0.2265	.7973

Note. Model terms tested with Satterthwaite testMethod.
Note. The following variables are used as random effects grouping factors: 'ID', 'Video_id'. *Note*. Type III Sum of Squares

Model summary

Fit statistics

Deviance (REML)	log Lik.	df	AIC	BIC
15198.5129	−7599.2565	20	15238.5129	15361.4239

Note. The model was fitted using restricted maximum likelihood. Please note that models with different fixed effects cannot be compared when REML is used. To use ML, switch 'Test method' to 'Likelihood ratio tests'.

Sample sizes

	Levels of RE grouping factors	
Observations	ID	Video_id
3448	96	36

Fixed Effects Estimates

Term	Estimate	SE	df	t	p
Intercept	−0.8368	0.9636	94.3111	−0.8684	.3874
Age	0.0038	0.0142	90.9965	0.2653	.7914
Sex (1)	−0.1901	0.1155	90.9981	−1.6459	.1032
Group (1)	0.0628	0.1177	93.9468	0.5334	.5950
Demand (1)	−0.7560	0.1986	30.0831	−3.8066	.0006
Demand (2)	1.5656	0.1696	30.0767	9.2321	< .0001
Context (1)	−0.5521	0.1353	30.0044	−4.0799	.0003
VHI (1)	−0.2026	0.1141	93.0348	−1.7765	.0789
Group (1) ∗ Demand (1)	−0.1031	0.0565	3310.2617	−1.8236	.0683
Group (1) ∗ Demand (2)	−0.0310	0.0482	3310.0429	−0.6419	.5210
Group (1) ∗ Context (1)	0.0278	0.0385	3310.0452	0.7222	.4702
Demand (1) ∗ Context (1)	0.3860	0.1985	30.0109	1.9445	.0613
Demand (2) ∗ Context (1)	−0.1281	0.1695	30.0045	−0.7561	.4555
Demand (1) ∗ VHI (1)	−0.2812	0.0561	3310.2072	−5.0159	< .0001
Demand (2) ∗ VHI (1)	0.1860	0.0478	3310.0412	3.8927	.0001
Group (1) ∗ Demand (1) ∗ Context (1)	−0.0237	0.0565	3310.0514	−0.4191	.6752
Group (1) ∗ Demand (2) ∗ Context (1)	−0.0154	0.0482	3310.0452	−0.3194	.7495

Note. The intercept corresponds to the (unweighted) grand mean; for each factor with k levels, k - 1 parameters are estimated with sum contrast coding. Consequently, the estimates cannot be directly mapped to factor levels. Use estimated marginal means for obtaining estimates for each factor level/design cell or their differences.

Linear Mixed Models_Demand

ANOVA Summary

Effect	df	F	p
Age	1, 91.00	0.0704	.7914
Sex	1, 91.00	2.7090	.1032
Group	1, 93.95	0.2845	.5950
Demand	2, 30.08	42.6359	<.0001
Context	1, 30.00	16.6459	.0003
VHI	1, 93.03	3.1560	.0789
Group ∗ Demand	2, 3310.17	2.7556	.0637
Group ∗ Context	1, 3310.05	0.5215	.4702
Demand ∗ Context	2, 30.00	1.8906	.1685
Demand ∗ VHI	2, 3310.14	14.4523	<.0001
Group ∗ Demand ∗ Context	2, 3310.05	0.2265	.7973

Note. Model terms tested with Satterthwaite testMethod.

Note. The following variables are used as random effects grouping factors: 'ID', 'Video_id'. *Note*. Type III Sum of Squares

Estimated Marginal Means

					95% CI	
Row	Demand	Estimate	SE	df	Lower	Upper
1	HD	−1.3400	0.2724	∞	−1.8739	−0.8062
2	LD	0.9816	0.2052	∞	0.5793	1.3838
3	MD	−1.3935	0.2849	∞	−1.9520	−0.8350

Warning: Estimation of degrees of freedom disabled (i.e., asymptotic results shown), because the number of observations is large. To force estimation, check corresponding option.

Note. Results are averaged over the levels of: Sex, Group, Context, VHI.

contrasts

	Estimate	SE	df	95% CI Lower	95% CI Upper	z	p†
HD vs LD	−2.3216	0.3076	∞	−2.9245	−1.7187	−7.5472	<.0001
HD vs MD	0.0535	0.3657	∞	−0.6632	0.7702	0.1462	1.0000
LD vs MD	2.3751	0.3188	∞	1.7503	2.9999	7.4501	<.0001

† P-values are adjusted using Bonferroni adjustment.

Linear Mixed Models_Context

ANOVA Summary

Effect	df	F	p
Age	1, 91.00	0.0704	.7914
Sex	1, 91.00	2.7090	.1032
Group	1, 93.95	0.2845	.5950
Demand	2, 30.08	42.6359	<.0001
Context	1, 30.00	16.6459	.0003
VHI	1, 93.03	3.1560	.0789
Group ∗ Demand	2, 3310.17	2.7556	.0637
Group ∗ Context	1, 3310.05	0.5215	.4702
Demand ∗ Context	2, 30.00	1.8906	.1685
Demand ∗ VHI	2, 3310.14	14.4523	<.0001
Group ∗ Demand ∗ Context	2, 3310.05	0.2265	.7973

Note. Model terms tested with Satterthwaite testMethod.

Note. The following variables are used as random effects grouping factors: 'ID', 'Video_id'. *Note*. Type III Sum of Squares

Estimated Marginal Means

					95% CI	
Row	Context	Estimate	SE	df	Lower	Upper
1	BUILT	−1.1361	0.2329	∞	−1.5926	−0.6796
2	NAT	−0.0319	0.2018	∞	−0.4274	0.3635

Warning: Estimation of degrees of freedom disabled (i.e., asymptotic results shown), because the number of observations is large. To force estimation, check corresponding option.

Note. Results are averaged over the levels of: Sex, Group, Demand, VHI.

contrasts

				95% CI			
	Estimate	SE	df	Lower	Upper	z	p†
BUILT vs NAT	−1.1041	0.2706	∞	−1.6346	−0.5737	−4.0799	<.0001

† P-values are adjusted using Bonferroni adjustment.

Linear Mixed Models_Demand*VHI

ANOVA Summary

Effect	df	F	p
Age	1, 91.00	0.0704	.7914
Sex	1, 91.00	2.7090	.1032
Group	1, 93.95	0.2845	.5950
Demand	2, 30.08	42.6359	<.0001
Context	1, 30.00	16.6459	.0003
VHI	1, 93.03	3.1560	.0789
Group ∗ Demand	2, 3310.17	2.7556	.0637
Group ∗ Context	1, 3310.05	0.5215	.4702
Demand ∗ Context	2, 30.00	1.8906	.1685
Demand ∗ VHI	2, 3310.14	14.4523	<.0001
Group ∗ Demand ∗ Context	2, 3310.05	0.2265	.7973

Note. Model terms tested with Satterthwaite testMethod.

Note. The following variables are used as random effects grouping factors: 'ID', 'Video_id'. *Note*. Type III Sum of Squares

Estimated Marginal Means

						95% CI	
Row	Demand	VHI	Estimate	SE	df	Lower	Upper
1	HD	YES	−1.8239	0.3082	∞	−2.4280	−1.2197
2	LD	YES	0.9649	0.2444	∞	0.4858	1.4440
3	MD	YES	−1.5010	0.3194	∞	−2.1271	−0.8749
4	HD	NO	−0.8562	0.2947	∞	−1.4338	−0.2787
5	LD	NO	0.9982	0.2298	∞	0.5479	1.4486
6	MD	NO	−1.2861	0.3062	∞	−1.8863	−0.6858

Warning: Estimation of degrees of freedom disabled (i.e., asymptotic results shown), because the number of observations is large. To force estimation, check corresponding option.

Note. Results are averaged over the levels of: Sex, Group, Context.

contrasts

	Estimate	SE	df	95% CI Lower	95% CI Upper	z	p†
HD: YES vs NO	−0.9677	0.2587	∞	−1.4747	−0.4606	−3.7405	.0006
LD: YES vs NO	−0.0333	0.2379	∞	−0.4996	0.4331	−0.1399	1.0000
MD: YES vs NO	−0.2149	0.2586	∞	−0.7217	0.2919	−0.8312	1.0000

† P-values are adjusted using Bonferroni adjustment.
